# Enhancing reproducibility in neuroimaging with containerized archival

**DOI:** 10.1007/s11682-026-01191-1

**Published:** 2026-08-24

**Authors:** Michael E. Kim, Praitayini Kanakaraj, Yihao Liu, Trent Schwartz, Zhiyuan Li, Nancy R. Newlin, Chenyu Gao, Kurt G. Schilling, Shunxing Bao, Baxter P. Rogers, Bennett A. Landman, Karthik Ramadass

**Affiliations:** 1 Department of Computer Science, Vanderbilt University, Nashville, TN USA; 2 Department of Electrical and Computer Engineering, Vanderbilt University, Nashville, TN USA; 3 Department of Radiology and Radiological Sciences, Vanderbilt University Medical Center, Nashville, TN USA; 4 Vanderbilt University Institute of Imaging Science, Nashville, TN USA; 5 Department of Biomedical Engineering, Vanderbilt University, Nashville, TN USA; 6 Department of Psychiatry and Behavioral Sciences, Vanderbilt University Medical Center, Nashville, TN USA

**Keywords:** Neuroimaging, Reproducibility, Containerization

## Abstract

Ensuring reproducibility in scientific research is crucial for validating findings, advancing knowledge, and fostering trust within the scientific community. Containerization of code is a widespread practice to help ensure reproducibility in neuroimaging research. However, there exists no universal method of sharing containers, nor is it feasible to broadly enforce rules due to the diversity of research practices. We posit that container sharing should include 1.) public availability, 2.) self-contained documentation, and 3.) procedures to ensure consistency of the container and its expected outputs. We demonstrate an approach for publicly releasing three separate containers using Zenodo. Our proposed method fulfills the design criteria for container sharing while incurring minimal overhead. As there are several tools available to distribute and maintain containers already, we discuss available alternatives.

**Supplementary Information:**

The online version contains supplementary material available at https://doi.org/10.1007/s11682-026-01191-1.

## Introduction

Reproducibility is an essential aspect of scientific research, as the generalizability of findings and results hinge upon the ability to recreate results in a controlled experimental setting (Niso et al., 2022). Despite seeming a simplistic matter of documentation, reproducibility in the field of neuroimaging is a challenging issue that requires attention, especially long-term reproducibility (Botvinik-Nezer et al., 2020; Munafò et al., 2017; Li et al., 2024). The two main components of ensuring reproducible science are the data used in the experiments and the methods for the experimental design. Different terms are used when referring to re-use or recreation of these two components, although the terminology can change depending on the particular scientific discipline (Barba, 2018). For the remainder of this paper, we will follow the definitions in Niso et al. (2022), where “replicability” specifies the use of new data with the same methods and “reproducibility” refers to the scenario when the same data and same methods are used.

While reproducing results with the same data is important, here we will only discuss the methodology component of ensuring reproducible science as it relates to the field of Neuroimaging and the broader domain of Computational Science. Neuroimaging methods are mostly code-based, and there exist several well-cited packages and libraries that are common in the field (Jenkinson et al., 2012; Tustison et al., 2014; Tournier et al., 2019; Garyfallidis et al., 2014; Fischl, 2012; Cox, 1996). The practice of sharing software or code with version control has also been facilitated by package managers and code repositories (pip · PyPI, 2024; GitHub, 2024; Anaconda Documentation — Anaconda documentation, 2024; Bommarito & Bommarito, 2019). Many academic journals or conferences in the Neuroimaging space are either requiring or suggesting that a section or statement regarding code availability is included in a, submitted manuscript, especially those with high impact scores (Paper Submission and Rebuttal Guidelines, 2024; Radiology | Instructions for Authors, 2024; Reporting standards and availability of data, materials, code and protocols | Nature Portfolio, 2024; Submission Guidelines | Scientific Data, 2024). These factors continue to push the field towards more reproducible and replicable research.

However, this vast number of different software packages and codebases introduce additional challenges that replicable and reproducible science faces. There is no guarantee that a researcher attempting to replicate methods will install the same version of software, as there may be compatibility issues across packages, libraries, or operating systems. A common way to circumvent such issues to better ensure reproducibility is virtualization or containerization (Kurtzer et al., 2017; What is a Container? | Docker, 2024; Bernstein, 2014). These virtual machines and containers can run a completely separate operating system than the host machine, allowing installation of previously incompatible software or libraries in an encapsulated environment. Furthermore, they can be transferred and shared so that multiple researchers can use the exact same code base without having to meticulously install particular versions of each dependency. Containers help separate human input factors from computational input factors in this way, as they prevent variety of versioning or design choices that could alter results. Historically, variability from running image processing pipelines on separate computational environments would not impact scientific findings, as the variability from large biological effects would dominate over any processing variability (Jäncke et al., 2015; Raz et al., 2005) (Supplementary Fig. 1). However, as studies become larger and target smaller effect sizes (Hibar et al., 2016), processing variability is no longer eclipsed by the biological effects researchers are trying to discover. Thus, in data processing for large scale studies it is essential to minimize the potential variability in data results, including image processing variability. In particular, containers have become increasingly popular due to their lightweight nature and portability (Potdar et al., 2020). The most common methods of containerization for neuroimaging research include Singularity (Kurtzer et al., 2017) (now commonly referred to as Apptainer, the current name of the original open-source Singularity project), and Docker (What is a Container? | Docker, 2024).

Despite the advances that containers bring to replicable and reproducible research, enforcing standardized sharing of containerized code is difficult. Solely sharing a recipe file used to build the container is problematic, as imported dependencies or codebases can change (ShabaniMirzaei, 2024). For example, a URL that hosts a dependency codebase could be modified without proper version control or become defunct overtime. Thus, a “rebuilt” container from the same recipe file may not have the exact same code as when it was initially built, or the build may not even be possible. Release of the container image itself can vary widely, from extensive documentation practices with robust version control and archival, such as fMRIPrep (Esteban et al., 2020), to a single link hosted on a private website. While releasing a container on an institution or lab website may be the easiest sharing method for the researcher, it can lead to longevity issues down the road. The institution may terminate their hosting service or the researcher responsible for maintaining the website may fail to keep it operational. Furthermore, even hosting containers on well-frequented container registries can cause issues if the repository is moved, renamed, deleted, or altered without proper version control (Opdebeeck et al., 2023).

In addition to the release of the container image, there are differences in the amount of metadata information provided with the container itself to supplement the code. This metadata information can include, but is not limited to, how to run the container, the expected inputs and outputs, and the date when the container was built. Such documentation is important, as it is very possible overtime that the original creator of the container is either no longer available or the repository that hosts the container becomes neglected (Lin et al., 2020). Furthermore, in fast-paced research environments, containers are often built in sandbox mode for rapid deployment and testing. This approach prevents the need to rebuild containers from scratch each time a new dependency is added or altered, especially when needing to repetitively rebuild early layers in a Docker image. However, the absence of a recipe file removes a lot of inherent documentation for how the container was built. Regardless, researchers releasing containers to the public may not wish to remake their container with a recipe file or may not have recorded the steps they used to create the initial container.

Medical imaging file formats evolve overtime, and thus, the expected inputs may require a specific extension or format. Volumetric images themselves can come in different formats, such as Digital Imaging and Communications in Medicine (DICOM) (Mildenberger et al., 2002), Neuroimaging Informatics Technology Initiative (NIFTI) (2024), Nearly Raw Raster Data (NRRD) (2024), the ‘.SRC’ extension used by DSI Studio (2024), MATLAB’s “matfile” (2024), *numpy* arrays (2024), etc. Associated metadata text files, such as BVAL or BVEC files for diffusion-weighted magnetic resonance imaging (dMRI) that indicate the strength and direction of the applied diffusion-weighting gradients during the scan, can have different text formats as well (Garyfallidis et al., 2014). While there exist initiatives such as Brain Imaging Data Structure (BIDS) (Gorgolewski, n.d.) that push to standardize the format of neuroimaging data, it is also difficult to enforce usage of specific file formats. Related to the file formatting is the data format, such as the orientation (e.g. Left-Anterior–Superior (LAS) vs Right-Anterior–Superior (RAS)), that was used for the medical imaging data files when creating the container for use. These expectations of data format for containers add another important documentation complication, as deviations from these expectations can lead to materially different outputs despite identical computational environments.

Another core aspect of replicable or reproducible research is ensuring consistency of the shared container., This issue ties closely with the documentation and container release, as other researchers who wish to run the algorithm in the container must make sure that it performs as expected. For example, if an available container is downloaded and then later modified and passed onto unknowing collaborators or lab members, it may no longer work as expected. Any researcher using the container needs to be sure precisely which algorithms and code are being run. Having safety nets to help confirm the validity of the container can help ensure container consistency. In the most advanced cases, continuous integration (CI) and continuous delivery (CD) tools are incorporated into the archival for automated release of new container versions (Belmont, 2018; Product and Features, 2024). CI/CD pipelines can also include functionalities like dependency checks or test cases to further ensure consistency of the container. One key detail to note is that there already exist popular methods for container archival and extensive CI (How to build a CI/CD pipeline with GitHub Actions in four simple steps - The GitHub Blog, 2024; Automated Software Delivery with GitLab | GitLab, 2024; Docker Hub Container Image Library | App Containerization, 2024). However, almost all of these provide some limit on either the allotted computational resources for CI/CD tasks or the amount of storage space, with capacity extensions available at a premium (Pricing · Plans for every developer, 2024; Pricing | GitLab, 2024a).

While there are certainly similarities between sharing raw code and sharing containerized workflows, containerization introduces specific peculiarities that can complicate reproducibility and replicability. When raw source code is shared, users can typically inspect the files line-by-line to understand the execution flow and troubleshoot errors. Conversely, containers act as fully encapsulated environments with their own internal directory structures (Kurtzer et al., 2017; What is a Container? | Docker, 2024). For containers without a defined entry point (e.g., a "runscript"), identifying the intended command to be executed can be difficult without explicit instructions., This issue can compound if pipelines contain compiled binary executables that are no longer human-readable. Together, these container-specific traits transform standard code-sharing considerations into threats to replicable and reproducible science, necessitating proactive documentation and rigorous archival practices prior to release.

Although containerization substantially reduces many sources of computational variability, it should not be viewed as a complete solution for reproducibility. Simply releasing a container does not guarantee that future researchers will be able to obtain the same executable image, understand how it should be used, or verify that it behaves as intended. The remainder of this work therefore proposes three minimum design criteria that we argue should accompany the public release of containerized neuroimaging workflows, along with a demonstrated method of how these design criteria can be fulfilled.

## Design criteria for container sharing

**Fig. 1:**
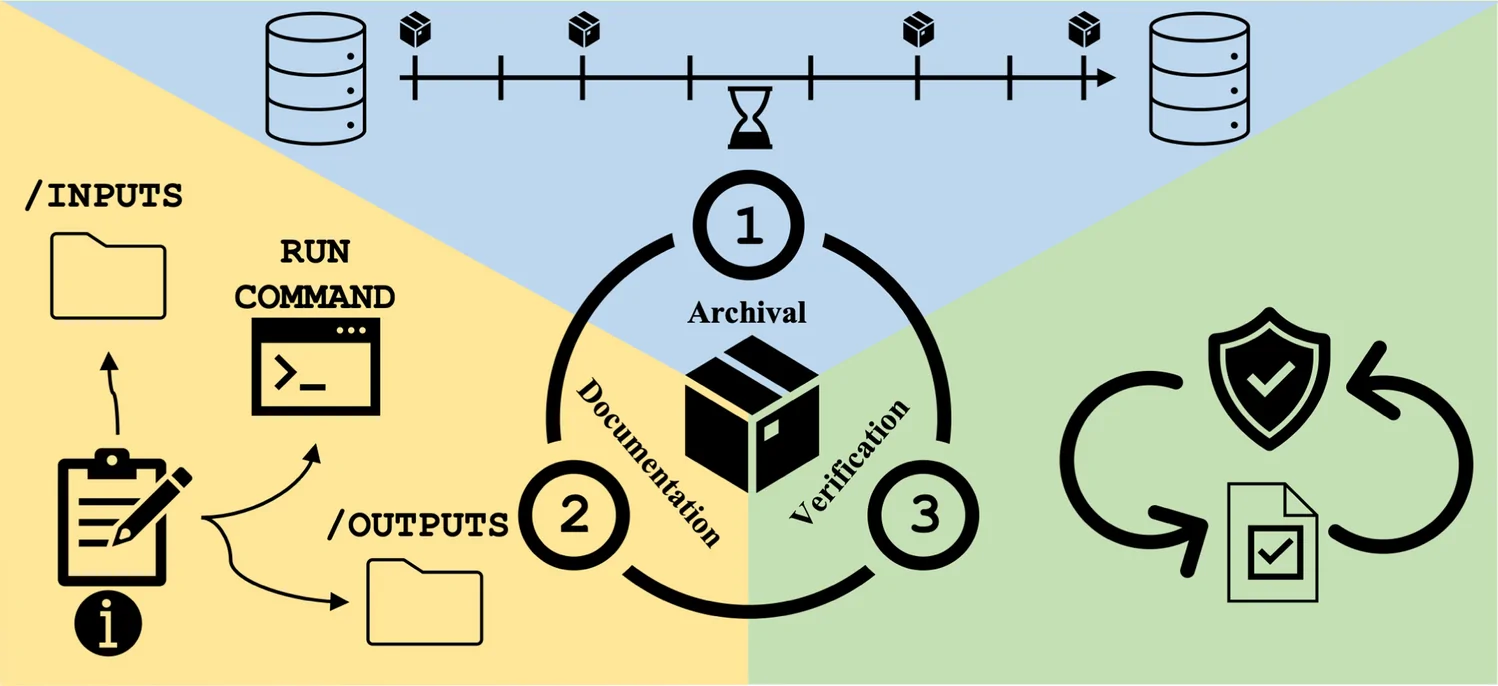
Containerization is a practice meant to promote reproducible and replicable science; however, simply giving another researcher a container cannot ensure this, nor do any standards exist for sharing containers. There are three considerations that must be taken into account for any container release: 1.) The container should be publicly accessible on an archival service that can outlast the utility of the container itself. 2.) Sufficient documentation should be included along with the container that can allow users to understand how to properly run the container, such as the command(s) and structure/format of inputs. 3.) There must be some aspect of consistency or verification so that the container algorithm will result in reproducible outputs

**Fig. 2:**
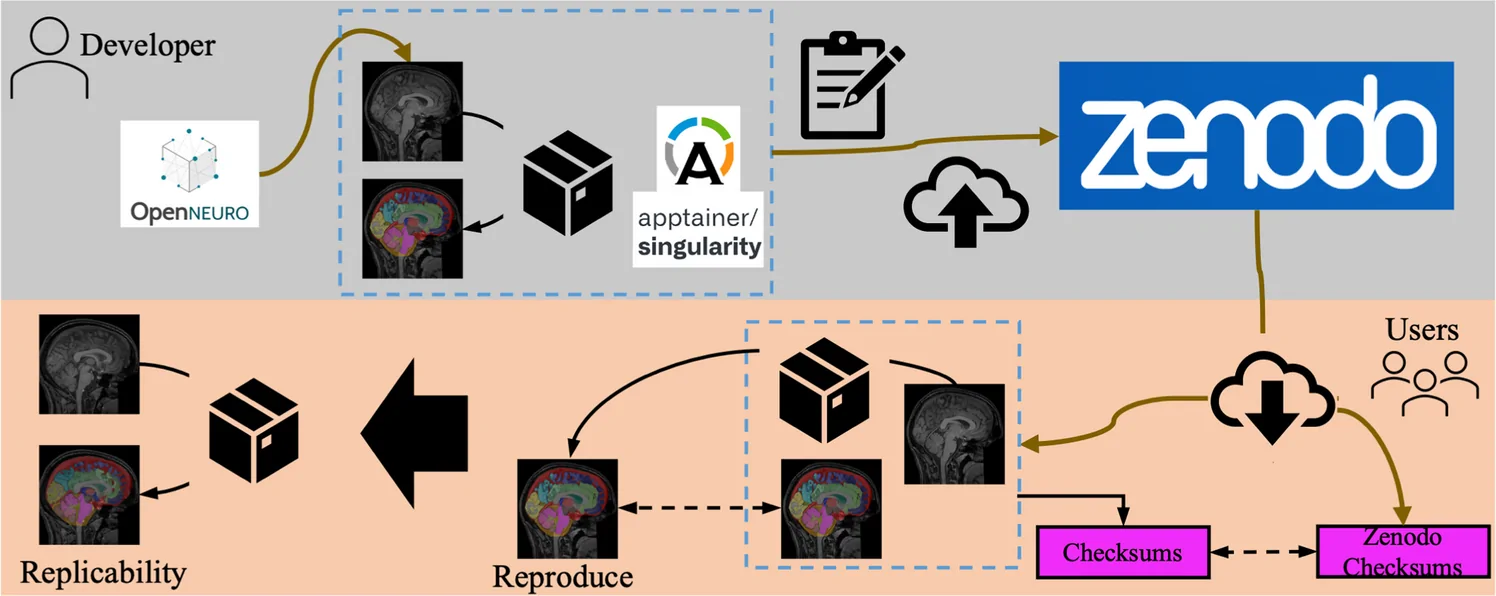
We utilize a publicly available archival tool and provide supplementary verification checks to demonstrate release of containerized code in a manner that promotes reproducible and replicable science. (Top) To help ensure consistency, we download open-source example test data from OpenNeuro and run them through our code, which has been containerized as a Singularity image. We then upload the outputs, inputs, and the container image to Zenodo, the archival method we use, and document how to run the container along with other information that potential users may find helpful. (Bottom) Once the container is uploaded, other researchers can download the test files and container along with checksums of all files that are provided by Zenodo. After comparing the reference checksums to those of the downloaded files for consistency, users can run the provided test inputs through the container and compare their outputs to the provided test outputs, hopefully reproducing the uploaded results. Upon successfully reproducing results, the users can then run their own data through the container, thereby replicating the original methods with their own data

Because of the mentioned vulnerabilities, container sharing requires more deliberate thought to ensure long-term reproducibility and replicability. However, there remains little guidance on what constitutes a minimally sufficient public release of a container or the practices needed to support long-term reproducibility. In particular, comparatively little attention has been given to defining the minimum information and safeguards that should accompany the public archival of a containerized neuroimaging workflow. Thus, in this paper, we first present an open method for releasing a container publicly that address the above concerns (Fig. 1). We pilot this method with three containers that are part of a large-scale processing pipeline maintained by our group (Kim et al., 2024a). We argue that the method should include the following design criteria for a bare minimum release of a container: 1.) making the container available for anyone to obtain on a publicly accessible archival service; 2.) self-contained documentation on how to run the container, such as the structure of inputs and outputs as well as the command(s) to run; and 3.) ensuring that there is some aspect of consistency/verification in the container so that the containerized algorithm provides reproducible results (Table 1). Our method is designed to be one that any researcher can adhere to, and we illustrate how our approach fulfills the above design criteria (Figs. 2, 4). Following our example, we discuss the pros and cons of other alternative options available to researchers, as we expect that strong preferences exist across labs and institutions, especially when containers are well-used and frequently maintained.

**Table 1:** Design criteria for sharing containerized code and methods

Design Criteria	Description
Documentation	When researchers wish to run a set of containerized methods or code, there are certain requirements, conditions, or expectations that must be met for the container to function properly, along with the command that is used to run the container. There may also be multiple functionalities, options, or flags that can be used. Equally important is specifying the current version of the container to prevent confusion about which set of code is being containerized. Conveying this information in an easily accessible manner is important for replicability and reproducibility of research. Additionally, details such as resource utilization or container build information can be helpful for troubleshooting problems
Public Archival	One of the most crucial aspects of publicly sharing a container is ensuring it is easily accessible to any researcher that wishes to replicate or reproduce methods from another researcher. The method used to store and disseminate the container should not only make the container openly available, but it should outlast the utility of the container itself and be able to properly support archival and distribution of the container
Consistency/Verification	Safeguards or checkpoints should be employed before using containerized code to make sure that the algorithms and code are running as expected; as the releasers of a container should be familiar with its execution, they are the ones that should make sure such methods of verification and consistency are available to other interested researchers

**Fig. 3:**
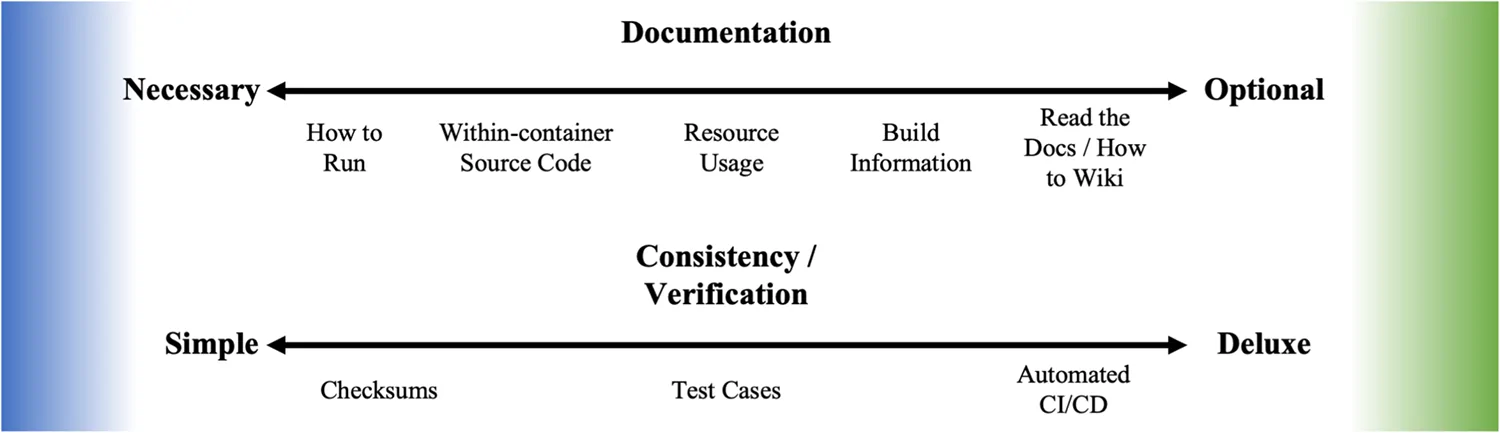
For practices of documentation and consistency/verification, the options are not mutually exclusive. In the most optimal scenario, every potential option would be included; however, releasing researchers must weigh the cost–benefit ratio of exploring each option, especially when the container is planned to be released “as-is” (no further modifications) in order to make research methods publicly available

While public archival is the most well-defined design criterion, both the documentation and consistency/verification design criteria exist along a broader spectrum of acceptable practices (Fig. 3). For documentation, we argue that a minimally acceptable release must provide details regarding expected input and output data formats, which includes any metadata, format, file naming, or units, as well as explicit instructions on the command(s) used to appropriately run the container. Similarly, baseline consistency verification requires an image checksum to ensure file integrity. More extensive practices are always highly recommended, not to mention beneficial, when possible or appropriate. Since implementing the highest tiers of this spectrum can require significant time and computational resources, researchers must evaluate the cost–benefit ratio based on the intended lifespan and utility of their specific pipeline. We explore the detailed implementations, alternative options, and practical trade-offs of these varying levels of rigor in the subsequent sections.

## Demonstrated example method

Our demonstrated method uses an open and publicly accessible archival service, Zenodo, that provides many opportunities for documentation, improving consistency, and ensuring longevity. While several platforms can satisfy these requirements (as discussed further in the subsection titled, “Alternative Options”), Zenodo provides an accessible infrastructure that readily supports public availability, comprehensive documentation, and consistency checks. The choice of Zenodo is intended to illustrate one practical implementation rather than imply that it is the preferred or only solution. Our three demonstrations of this container sharing method can be found here: https://zenodo.org/records/14593034, https://zenodo.org/records/14586026, https://zenodo.org/records/14618566.

### Containers

While there exist many different formats for containers, we use Singularity containers as our containerization method. Our choice is twofold: first, Singularity images can be run on high-performance computing (HPC) clusters, which we use to process our MRI data. Second, if there are bugs in any of the pipelines, the containers can be converted to a sandbox mode where fixes or alterations can be made and tested without having to remake the entire container. For containers that exist in layered formats, such as Docker, bugs that exist in very early layers would require most of the container to be rebuilt. This could potentially cause a significant issue if necessary dependencies in later layers have since become defunct. We select three different containers used in our large-scale neuroimaging pipeline (Kim et al., 2024a, b): PreQual, used for dMRI preprocessing (Cai et al., 2021, 2025); a container for registration of a structural atlas with a T1-weighted (T1w) template to a dMRI scan (Kim, 2025); and SLANT-TICV, a brain segmentation algorithm (Liu et al., 2022, 2025).

### Public archival

**Fig. 4:**
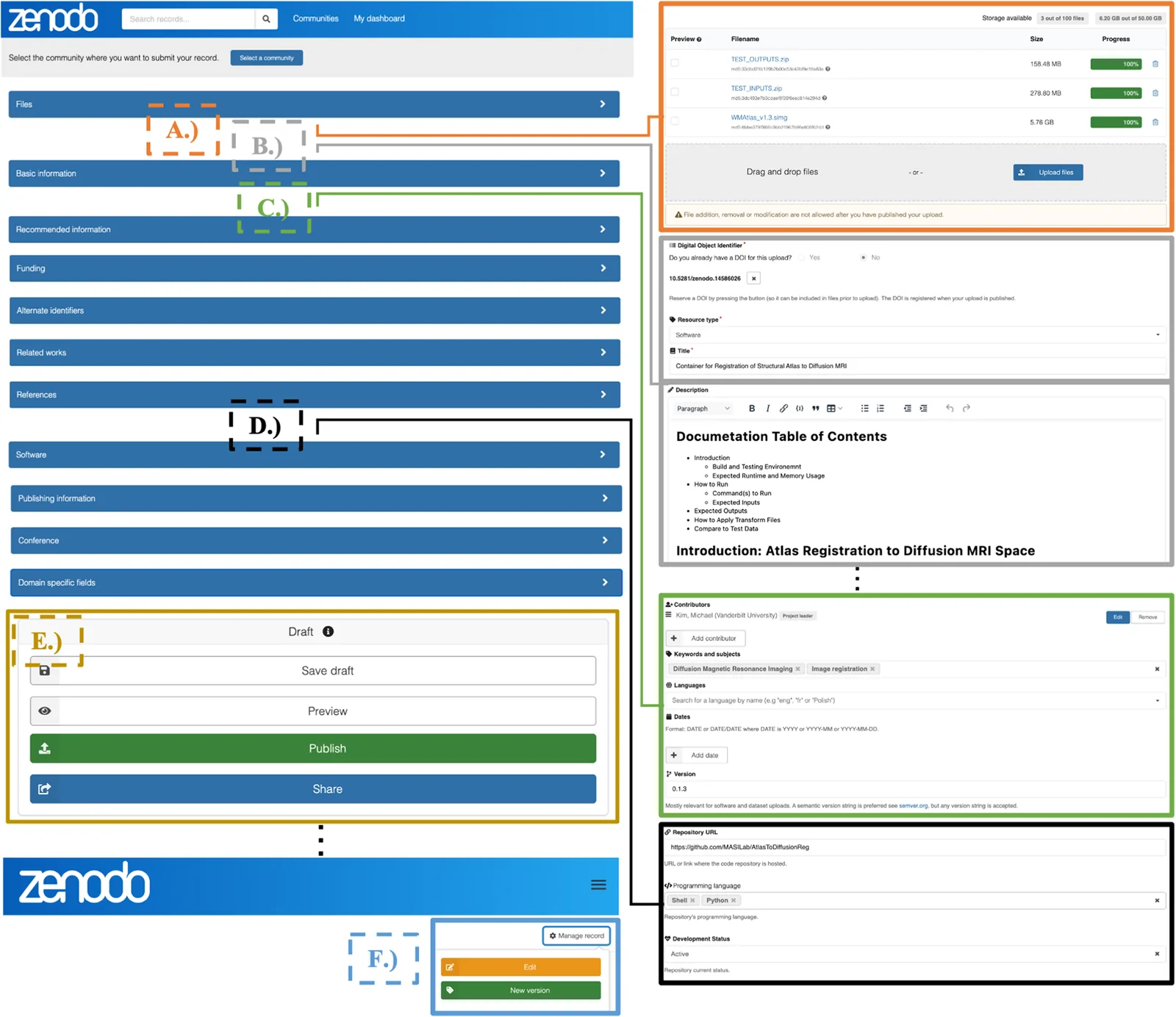
We demonstrate release of containers on Zenodo, which is a process replicable by any researcher. **A** Multiple files can be uploaded per release, such as the container image and test data, up to a limit of 100 files and 50 GB. **B** A DOI can be automatically generated for the release, and other information can be specified as well, such as the title, type of resource, and a description tag that can serve as a field for documentation. For our example releases here, we have included documentation such as the build information, resource requirements, how to run the container, descriptions of the outputs, how to compare to testing data, etc. **C** Keywords can be added to make the container more searchable, and version information can be included to indicate updates if it is a subsequent release of the container. **D** A repository with the source code for the container can also be linked to the Zenodo page. **E** The release can be saved as a draft or previewed before the container is publicly released. **F** If the container is updated, a newer version can be released. An existing release can be edited ONLY with regard to the documentation; files can neither be modified, added, or deleted. Note that there are other provided information fields the user can fill out as well, such as funding, references, publishing information, etc. if relevant

For sharing the singularity images, we choose to use an archival tool hosted by the European Council for Nuclear Research (CERN) known as Zenodo, a platform created for sharing and preserving open research, especially the vast number of smaller projects that form the “long-tail of science” (Nowak et al., n.d.). Our selection of Zenodo as the achival method for container sharing is based on the design criteria described above. First, Zenodo is openly accessible and allows for extensive documentation. It also has a relatively high storage capacity of 50 GB per version. Users can provide additional updated versions of containers or software, permitting user-controlled versioning, and can import duplicate files from previous releases without re-uploading. Each release is assigned its own DOI, facilitating communication over which version was used for a paper or study and making it citable in a scientific manuscript. Additionally, once a file is uploaded to Zenodo, it cannot be removed from the website except for extreme cases with legal or ethical concerns. This inability to delete means that it is very unlikely other researchers will be unable to find a previously released version due to updates or modifications from the releasing author. Finally, Zenodo takes several actions to preserve longevity of data integrity, such as multiple backups and checksums (Policies | Zenodo, 2025). For these reasons, we feel Zenodo is ideal for researchers intending to release a container with minimal effort. We illustrate the process of uploading to Zenodo in Fig. 4.

### Documentation

There is a spectrum of options for documentation of containers, ranging from essential information that allows users to properly run the encapsulated algorithms to more supplementary knowledge that can be helpful with potential debugging (Fig. 3). We use several of these documentation practices for releasing our containers, with the information available either on a linked GitHub page that contains all source code or directly on Zenodo. First, we specify the expected format for the inputs to the container, which not only includes the volumetric image format, but also the expected naming of the files and text format for metadata files such as the BVAL and BVEC files. We also provide example commands for how to run the containers in different scenarios, as well as expected memory usage for the container and what each output file contains. Even though they are not essential for running the containers themselves, we provide build information for the containers in the form of Singularity recipe files on the GitHub page so that users can readily see the necessary dependencies and software versions. Finally, we provide all original source code within the container itself. As the GitHub page for PreQual already contains extensive documentation, duplicating the entirety of this information on an archival site is impractical. Instead, we use PreQual as an example of how containers can be released on Zenodo while incorporating alternative documentation methods yet keep everything synchronized. In this instance, we provide essential summary information directly on Zenodo and ensure the stable Zenodo release points directly to the documentation on GitHub, establishing a consistent and reliable source of truth across platforms.

While these containers are not explicitly released in the BIDS-App framework, they can be run with BIDS-organized datasets, a process which we continue to follow as we maintain our processing pipelines (Kim et al., 2024a). The ability to run containers with BIDS compliance is very important, as the BIDS organizational standard helps ensure consistency of data in the neuroimaging community (Gorgolewski et al., 2016). In our documentation, we provide instructions for how to run these containers as BIDS-Apps with regard to the expected organizational structure of the input and output data. We strongly encourage providing these instructions for any container release whenever possible or applicable.

### Consistency/verification

Though we provide information how to run the containers and what the outputs should look like, we also take steps to ensure that there are no issues with how the containers themselves run on different hardware with different users. Similar to documentation practices, there is a range of methods available for verifying the integrity and functionality of containers (Fig. 3). The first step is to ensure that the container images are not corrupt. For this, Zenodo provides an md5 checksum for every file that researchers can compare their downloaded image to. Thus, even if a container image is altered by a collaborator or was not downloaded properly, there is a single command line sanity check to make sure that the expected code is being run. An additional consistency check that we include with the Zenodo upload is a tutorial with example test input data and output data, derived from a session of the AOMIC ID1000 dataset (AOMIC-ID, 1000 - OpenNeuro, 2025; Snoek et al., 2021). We provide these files so that other researchers can not only have a small guiding demo for how to run the container but also some example outputs for comparison to make sure the container can be run as expected.

## Discussion

Previous researchers have also articulated concerns and considerations for containerization in fields other than Neuroimaging. For the Bioinformatics domain, Gruening et al. and Alser et al. both suggest a core set of views for sharing any type of software, which includes sustainability, reusability, minimal size, and transparency among others (Alser et al., 2024; Gruening et al., 2019). While the suggestions from both publications mostly align with the design criteria proposed in this manuscript, Gruening et al. and Alser et al. also suggest that the container build process should be reproducible. As mentioned earlier, researchers wishing to replicate methods should aim to obtain a pre-built and archived container image rather than rebuilding a new container image. This practice helps to avoid potential dependency issues upon rebuilding at a later time point.

Moreau et al. posit that sharing both a recipe file and the container is essential for reproducibility (Moreau et al., 2023). However, containers built in sandbox mode, often in fast-paced research environments for rapid deployment and testing, typically do not have associated recipe files. To help ensure that the “long-tail of science” is properly preserved, archiving the exact container image used in the methodology of a research paper is a more effective method for ensuring replicability, rather than writing a recipe file to try to rebuild the container from scratch. Moreau et al. also provide best practices for documentation, all of which are covered in the design criteria proposed in this manuscript.

One potential concern that Alser et al. and Moreau et al. both raise is that a learning curve exists for understanding how to use containers. Having sufficient documentation on how to run a container upon release for public archival can help mitigate any issues that arise from unfamiliarity with container images. Providing test cases and a tutorial with the container release can also help users become more familiar with running container images. For researchers unfamiliar with building containers, Neurodocker can serve as an instructional tool by generating example recipe files to build containers with commonly used neuroimaging software packages (Welcome to Neurodocker! — Neurodocker documentation, 2025).

Although we provide empirical analyses in the Supplementary Material that illustrate computational variability in both containerized and non-containerized workflows, these analyses are intended to motivate the importance of reproducibility and demonstrate the existence of container-specific reproducibility issues rather than provide a comprehensive empirical evaluation of the proposed design criteria. This manuscript instead focuses on proposing practical minimum design criteria and demonstrating one implementation that satisfies them. Further empirical investigation into the contribution of these design criteria across diverse neuroimaging workflows remains an important area for future work.

Above, we have outlined design criteria to support reproducibility in future releases of neuroimaging pipelines. We hope that providing this information, alongside our demonstrated approach for container release on Zenodo, will facilitate reproducibility of scientific results and distribution of image processing algorithms. The widespread adoption of standardized container archival practices, such as those proposed here, can provide the stable methodological foundation required to conduct future neuroimaging studies. Importantly, further empirical investigation into the impact of computational variability on biological effect sizes is needed to better inform the interpretation of both past and future research. However, we leave such analyses to future work, as they are beyond the scope of this manuscript.

Below, we discuss alternative methods for container archival, considerations for appropriate documentation approaches, and ways to ensure the consistency/verification of the container itself.

### Alternative options

Our method is broadly replicable with minimal overhead to facilitate using the shared code. Still, we understand there are several options available to researchers for fulfilling each of the design criteria. Here, we will discuss our methods alongside some of these alternative options, which is by no means an exhaustive list. A universal note is that increasing complexity or functionality generally also may incur higher costs, whether financial or in terms of time investment. We note that details on the different archival methods presented here reflect their current state as of Feb 3, 2025.

#### Archival

There exist several potential archival methods for container images (Table 2). However, it is important that whatever method being used to archive and share the containers will outlast the lifetime usability of the container itself. For example, Singularity Hub is one such container archival service that was terminated (Singularity Hub Documentation, 2024). While containers released on Singularity Hub are still available for download, no more containers, including updated versions of existing containers, can be archived using the service. Google Container Registry (gcr.io) is also deprecated at the time of this writing, with functionality transitioning to Google’s Artifact Registry (Container Registry deprecation | Container Registry documentation | Google Cloud, 2024). Also, when using methods that permit removal or altering of released containers, we urge researchers to not modify or delete their previous uploads to prevent replicability issues. Rather, if changes need to be made, the proper course of action is to release a new version of the container while keeping the older release available as is.

**Table 2:** Different archival options available for releasing/sharing containers

Archival Option	Zenodo	OSF	Docker Hub	Quay.io	BIDS-App	GitHub (ghcr.io)	GitLab Container Registry	For-Profit Container Registry	Cloud Storage
Storage Limit	50 GB; 200 GB for datasets; 100 file maximum	5 GB per file; 50 GB total per project	“Unlimited” with potential usage-based charges	Unlimited	Same as Docker Hub	500 MB	Unlimited	Limits depend on tier	Scalable freemium model
Storage Add-ons	None	Connect to Cloud Storage	Usage based charges *	Payment plans for private repositories	Same as Docker Hub	$0.008 per day per GB	N/A	Pay what you use	Available on payment
Consistency Support	md5 checksum	md5 and SHA256 checksum	SHA256 checksum	SHA256 checksum	CircleCI	SHA256 checksum; built in CI/CD	SHA256 checksum; built in CI/CD	SHA256 checksum	None
Container Types Supported	Any	Any	Docker	Docker, podman, rkt	BIDS-App Docker	Docker/OCI format	Docker/OCI format	Docker/OCI format	Any
Can Remove or Alter Existing Container	No	Yes, with history log	Yes	Yes	Yes	Yes	Yes	Yes	Yes

* Note that this is for public repositories, but private repositories have their own limits

Another consideration is to ensure that the container works prior to release, as many researchers may “release” their container in order to check a box for paper publication, but the container does not work as intended. For BIDS-Apps, the container is required to work with BIDS-organized data and pass test cases before it can be hosted as a BIDS-App (Gorgolewski et al., 2017); other potential repository services, such as cloud-based general storage services or Docker Hub (Docker Hub Container Image Library | App Containerization, 2024), do not have requirements to upload container images. In these instances, it is up to the original researcher to ensure consistency in the functionality of the container. There are also differences among archival services for uploading and downloading container images. Some services, like Zenodo, require uploading files, meaning containers in formats like Docker will need to be converted to tar balls using commands like *docker save* and then subsequently loaded with commands like *docker load*. Finally, as we focus on public release of containers for reproducibility and replicability, we will not discuss private or internal methods of container hosting that many archival systems also provide.

#### Zenodo

Given the extensive discussion of Zenodo in previous sections, further examination of its application will be concise. The advantages include the capability to upload any container type as a file, the provided DOI for each uploaded container, and the permanence of the container upload. Zenodo also allows for the inclusion of metadata documentation, such as references, associated publications, etc. that can enhance the discoverability of the repository through online searches. Records can be saved as drafts until the researcher is prepared to publish them, such as when the associated paper is published. Files released on Zenodo are preserved for the duration of the Zenodo repository’s existence. Currently, this duration corresponds to the lifetime of the host laboratory CERN, which is projected to be at least the next 20 years (Policies | Zenodo, 2025). However, Zenodo’s storage capacity is limited to 50 GB per project version, which may be insufficient for very large containers. Furthermore, Zenodo offers limited automation capabilities compared to other container archival solutions. Therefore, Zenodo may be most suitable for researchers who intend to release their methods and code without frequent updates to the container.

#### Docker hub

Docker Hub is a widely used container repository, as it serves as the default repository for Docker images and does not require payment by default (Docker Hub Container Image Library | App Containerization, 2024). Consequently, it has a large user base and is well-known among researchers. Interfacing with Docker Hub is efficient due to its command line interface (CLI) options for pushing and pulling Docker images. The implementation of *tags* in Docker images facilitates efficient version control, enabling users to view all previous named releases in container repositories. Docker Hub can also be integrated with source code repositories and CI/CD tools for automated builds and version control. A notable advantage of Docker Hub is the potential for unlimited free storage of publicly released containers for users.

Despite these advantages, Docker Hub has certain limitations. Recently, Docker Hub revised its policy on storage and activity, specifying:

*“When utilizing the Docker Platform, users should be aware that excessive data transfer, pull rates, or data storage can lead to throttling, or additional charges. To ensure fair resource usage and maintain service quality, we reserve the right to impose restrictions or apply additional charges to accounts exhibiting excessive data and storage consumption.” *(Usage and rate limits | Docker Docs, 2024)

This variable pricing policy implies that large or high-traffic containers may face limitations or require payment with little notice. It is important to highlight that this should not impact smaller projects with lower container image pull rates. However, Docker is the only supported image format, rendering Docker Hub incompatible with other container types. Furthermore, Docker Hub does not follow a policy of immutable releases like Zenodo, potentially resulting in missing or altered containers (Opdebeeck et al., 2023). Overall, Docker Hub is suitable for researchers with small container releases and also has the capability to support larger projects; It can reliably archive Docker images and meet the design criteria, provided that each subsequent upload has a unique tag and no uploads are deleted.

#### Quay.io

Quay.io is a container registry for Docker, podman, and rkt type containers (About Quay IO, 2024). Similar to Docker Hub, the registry supports pushing, building, and pulling containers via a CLI process, and provides an SHA256 checksum for ensuring container image integrity. Container repositories can be integrated with GitHub, GitLab, or Bitbucket for automated container building and CI/CD. A notable feature is the free and unlimited storage, without restrictions on the number of public repositories or pull/push traffic, unlike Docker Hub. However, the registry cannot support containers such as Singularity, and permits researchers to delete or alter their files. Nevertheless, it serves as a viable alternative to Docker Hub due to its similar functionality, particularly if the storage or traffic limitations become burdensome. Quay.io can fulfill the design criteria for archiving containers, provided that uploads are not modified or deleted.

##### OSF

The Open Science Framework (OSF) was launched in 2012 by the Center for Open Science (COS) as a tool to create and develop projects and preserve research workflows in a transparent manner (Foster & Deardorff, 2017). Researchers can design entire projects in an organized fashion, including the upload of files such as data, code, or container images. While OSF initially offered unlimited storage capacity with a 5 GB per-file limit, this has been reduced to 50 GB due to high usage over the past several years (Shared Investment in OSF Sustainability, 2024). Created for similar purposes, OSF and Zenodo share many similarities, such as the provision of a DOI for public repositories and the use of tags/categories that can be assigned to projects to increase searchability of methods. OSF also includes built-in version controlling, maintaining a historical record of all file and repository changes, which is recorded on the project page.

However, there are two significant distinctions that differentiate OSF from Zenodo. First, files and even projects can be deleted from OSF, with only the record of the file deletion to indicate a change. This includes instances when files, such as containers, are updated and replaced with newer versions. Second, both the individual 5 GB and project-level 50 GB limits on OSF can be circumvented by linking storage add-ons such as Amazon S3, OneDrive, Google Drive, etc (Storage Add-ons - OSF Support, 2024). Thus, while Zenodo is more dedicated to file preservation, OSF provides greater flexibility in storage capacity. Considering all factors, OSF offers an effective solution to researchers for open release of containers and can be used to satisfy the design criteria for container archival.

#### GitLab/GitHub container registries

GitHub and GitLab, both code repository tools, host their own container registries: ghcr.io and the GitLab Container Registry respectively. One of the advantages to these platforms is that CI/CD tools are built into the services, which facilitates methods of consistency/verification. Committing code to online repositories is a standard practice, leading to increased familiarity for researchers with either or both platforms. Moreover, using either ghcr.io or the GitLab Container Registry offers the advantage of a unified interface, enabling developers to manage their code and container images in one place. This integration can streamline workflows by eliminating the need to switch between tools and helping to ensure that repository updates and container versions are synchronized. Additionally, an SHA256 checksum for containers is also provided by both platforms.

Similar to many other container repositories, ghcr.io and GitLab Container Registry support only Docker and Open Container Initiative (OCI) compliant image formats. Furthermore, the free options for both registries are relatively limited compared to other alternatives. ghcr.io is a subset of the GitHub Packages Registry; for the Packages Registry, the free tier provides a maximum of 500 MB of storage with 1 GB per month of data transfer. Exceeding the allotted resources costs $0.008 USD per GB of storage per day and $0.50 USD per GB of data transfer, with a one-month grace period before charges are applied (About billing for GitHub Packages - GitHub Docs, 2025). Regarding storage, GitLab states for its container registry:

*“There is no explicit storage limit storage limitation, which means a user can upload an infinite amount of Docker images with arbitrary sizes. This setting should be configurable in future releases.” *(GitLab container registry administration | GitLab, 2025)

However, the free tier of GitLab provides only 400 min of compute time per month (Pricing | GitLab, 2024a). These limitations in the free tier of either tool can render large containers with long build times infeasible to archive. Researchers considering these platforms for archiving and releasing their containers should consider that ghcr.io and the GitLab Container Registry are potential options to facilitate consistent development of tools over time, particularly if the source code exists in a repository where a container can be conveniently built. Using either method can fulfill the design criteria for sharable containerization, so long as any necessary storage extensions are paid to prevent the container access from becoming defunct.

#### BIDS-App

The BIDS-App framework, developed by Gorgolewski et al., for archival of neuroimaging containers is a significant advancement to the neuroimaging field (Gorgolewski et al., 2017). Not only does it leverage existing tools such as Docker Hub, CircleCI, and GitHub, but it also has inherent compatibility with the BIDS organizational data format, facilitating the execution of multi-container pipelines and reducing potential disorganization in the format of input files. The creators of BIDS-App have also provided extensive documentation and examples on how to create a BIDS-App container. Along with dozens of released containers, these factors facilitate the release of documented and version-controlled containerized code through the BIDS-App framework.

Whenever possible, neuroimaging pipelines should be released as BIDS-Apps in order to enhance accessibility to the broader neuroimaging community. However, legacy tools or pipelines may have reproducibility concerns due to discontinued support. Consequently, they may not be BIDS compliant, and thus cannot be made into BIDS-Apps. While the neuroimaging community continues to develop more advanced and accurate tools, these legacy pipelines must still be maintained until they become obsolete. The BIDS organizational format does not currently officially support all types of neuroimaging data, such as susceptibility-weighted imaging, although extension efforts of BIDS to include some of these datatypes are in progress (Brain Imaging Data Structure, 2025). Pipelines for these currently unsupported datatypes may not be releasable as BIDS-Apps. Another category of imaging research methods that may not be archivable as BIDS-Apps includes those coming from multi-organ imaging studies (Yang, 2023; Yu, 2024; McCracken et al., 2022), as BIDS currently supports only neuroimaging datatypes. Additionally, researchers may wish to retain their current organizational structure for internal legacy purposes, such as longitudinal datasets that have existed for decades and continue to be updated (Ferrucci, 2008; Bao et al., 2022). Therefore, in these instances, BIDS-App may not be a feasible archival solution. Nonetheless, the BIDS-App framework adheres to the design criteria for container sharing, making it a highly attractive option for archival.

Note that for these instances where BIDS-App is not a feasible archival solution, the reproducible method we demonstrated is still consistent with BIDS-App principles of ensuring reproducibility. In both methods, test cases provide verification for the container. Instructions on how to run each pipeline on BIDS-formatted data are also provided, as BIDS-compliance is a requirement for BIDS-Apps. Additionally, the automated version control of BIDS-Apps and the ability to retrieve previous versions of BIDS-App containers are complemented by the permanent version releases available on Zenodo.

#### For-profit container registries (Amazon, Microsoft, Google)

Several for-profit companies offer container registry services, with the most notable being Google’s Artifact Registry (previously Google Container Registry), Amazon’s Elastic Container Registry (ECR), and Microsoft’s Azure Container Registry (ACR). All three are similar in general functionalities, with differences in storage capacity and pricing. Containers can be pushed and pulled from the command line, and storage expansion is generally not an issue since users can take as much as they need, provided they are willing to pay. Coming from large corporations, the parent companies of these registries also offer extensive user support, even during significant infrastructure changes, such as Google’s transition from the Google Container Registry to the Artifact Registry (Container Registry deprecation | Container Registry documentation | Google Cloud, 2024).

However, all three currently support only Docker or OCI-compliant image formats, although SHA256 checksums are provided for all containers. The images can also be modified or removed by the owner of the container, or even by the company if the owner fails to pay for storage or computation costs. For any sizeable container, ECR is the only option that could be used for archival on a free tier subscription plan, offering 50 GB free with $0.10 per additional GB per month (Fully Managed Container Registry, 2025). The Google Artifact Registry provides only 500 MB of free storage with $0.10 per GB per month of extra storage (Pricing | Artifact Registry | Google Cloud, 2025), and ACR does not have a free tier, with the lowest-priced tier costing $0.167 per day for 10 GB of storage and $0.00334 per day per GB for extra storage (Pricing - Container Registry | Microsoft Azure, 2025). Therefore, researchers who intend to release their containerized code for free should consider other options. However, any of these registries can be used to archive containers provided that existing versions of containers are not deleted or modified.

#### Cloud-based storage services

Storage services, such as Google Drive, OneDrive, and similar platforms, allow users to openly share files and may initially appear to be suitable options for researchers releasing container images. The effort required is minimal, as users only need to upload an image and provide a download link. Additionally, these services can support the upload of any file type, which means that any type of container can be uploaded as well. However, researchers should be aware of the limitations when using these types of services for public release of containers. First, other open options like Zenodo or OSF offer additional services that support reproducible research, whereas cloud storage services primarily provide storage. Moreover, the uploaded files are tied to the owner’s account; if the account or service were to become inactive or be deleted, the files may also be removed. While cloud-based storage services can be used to share containers, the platforms themselves do not inherently provide extensive support for ensuring container consistency, version control, or additional documentation. Thus, in order to fulfill the design criteria, researchers must supplement the services by manually including checksums, version numbers, and explicit documentation when releasing containers.

### Documentation

The boundaries for ‘alternative methods’ of documentation are not well-defined, as researchers can choose to document as much or as little as they deem necessary. However, there are several methods for providing documentation and specific practices to consider (Fig. 3). The methods discussed below do not need to be mutually exclusive; incorporating multiple documentation practices generally beneficial as long as all documentation is clear and up-to-date.

When providing documentation, a substantial amount of information could be included. Essential information should be provided as frequently and consistently as possible to help ensure that other researchers can use the container as intended. One of the most crucial pieces of information is how to run the container, which can be particularly important to detail if the container has multiple functionalities. This includes the shell command to execute and the expected inputs and outputs for each functionality, such as the file types or formats and an explanation of all the outputs. Resource information such as expected run time or CPU/GPU memory usage can be equally important to ensure that users are aware of the hardware requirements and expectations for running the container. Another frequently omitted category of information is the original system configuration that was used to test, build, and run the container before release. This information can be especially useful for containers that include deep learning or require dynamic compilation of code during runtime (‘just in time’ (JIT)), as is the case with many *PyTorch* extensions (Custom C++ and CUDA Extensions, 2024).

Although not explicitly designed for documentation purposes, a recipe file used to build a container serves as a useful record for understanding how a container was created, as it can facilitate debugging of potential issues by other researchers. As mentioned earlier, caution should be exercised when using recipe files to rebuild containers, since dependencies may have changed since the initial build. For reproducibility and replicability, it is generally safer to download and use a provided final container build. When containers are built using a sandbox method, an alternative to a recipe file is to document every command executed to set up the container and provide these steps as reference. Another option is to debug the setup process using a sandbox method and then use the documented steps to create a recipe file which can then be used to immediately rebuild the container. It is essential to then validate that the recipe build is identical to the sandboxed version.

Researchers may provide documentation inside the container itself in the form of README files, ‘–help’ flags, or in-line code comments. Within-container documentation is one of the most convenient methods because the notes will always be present with the container image. Even if external documentation exists in other places, such as a GitHub repository, read the docs, or documentation files distributed alongside the container, providing duplicates of all these files internally or links to these external sites offers additional assurance in case these external sources become defunct or lost. When hosting documentation in multiple locations, it is essential to ensure that all sources are synchronized to prevent outdated or contradictory information from causing confusion. Some external documentation sites, such as “Read the Docs”, allow for some synchronization through linking codebase repositories to the external documentation pages. One critical component to make available is the source code for compiled binaries, relevant for languages such as C/C + + or MATLAB, available within the container itself. This helps to ensure that users know exactly what version of the code was used to build the executable files.

For more extensive containers or projects, relying solely on within-container documentation may not be the most convenient documentation practice for other users. In these scenarios, external sources of documentation can be highly valuable, such as a GitHub repository, or, for very comprehensive containers, hosting a ‘Read the Docs’ or ‘How to Wiki’ similar to the fMRIPrep model (fMRIPrep, 2024; Esteban et al., 2018). An additional benefit of this approach is the professional appearance and increased searchability, which can appeal to potential users or be helpful if the container is applicable to a broad audience. Links to the external documentation sources can also be included within the container. However, it is important to consider the trade-off with increasing levels of documentation, particularly if the container release is meant to be a quick yet complete sharing of methodology. Regardless of the container size, releasing researchers should ensure that all necessary information for running and using the container is properly documented in a conveniently accessible location.

### Consistency/verification

It is important to note that there are several options for ensuring consistency in reproducing results when sharing containers, and they do not need to be mutually exclusive (Fig. 3). Implementing additional consistency checks is a good practice but setting up exhaustive automated CI/CD with several test cases can be time intensive. At a minimum, researchers should provide a checksum of the final container image upon release so that other researchers can verify the integrity of the container. Provided that there is sufficient documentation for running the container and all required code is inside the container (i.e. the container is not required to reference code that exists on the running workstation), a checksum can virtually guarantee that the algorithm is the same due to the low rate of collision for checksums. One reason Zenodo was utilized is that a checksum is provided on the published page; other archival sites, such as Docker Hub, also provide checksums.

Additionally, there are further consistency measures that can be included with little overhead, such as releasing a test case with the container. As demonstrated in our examples, including one or two examples of inputs and outputs from any of these datasets along with the container release can provide a straightforward additional check, allowing other researchers to verify that the code and algorithms are functioning as expected. The code in the container should have been already run by the releasing researcher, and several multi-modal neuroimaging datasets exist online that can be included as test cases without restrictions. Open access datasets can be found on Zenodo or OSF, and there are sites specifically for open public sharing of neuroimaging datasets, such as brainlife.io (Hayashi et al., 2024) or openneuro (Markiewicz et al., 2021). One consideration is to include the test data inside the container itself to provide ease of access, although this may not be feasible for some larger test data outputs, such as tractography, when using archival services that limit individual file sizes.

When providing instructions on how to run test data through the container, researchers should make sure to specify which parameters/hyperparameters are used, including specification of default settings, as others may have a specific method for running common algorithms. For instances when algorithms are non-deterministic, such as FSL’s EDDY, certain image registration procedures, or most tractography techniques, slight differences in outputs may still occur. In these cases, if releasing the test data, researchers should include a reminder regarding these potential output differences when comparing to the released testing data. Additional non-determinism checks, such as our assessment across multiple hardware configurations (Supplemental Figures S2–S4), may provide useful information for end users. However, while such checks can improve transparency, they are not strictly required for other researchers to run a container in a replicable manner.

For CI/CD, such as the functionalities provided by CircleCI, Git hooks, workflows in GitHub, or pipelines in GitLab, researchers can include test cases and other checks that are executed when any changes are made to the containers. These CI/CD tools can automate consistency checks, version control, and container release. These benefits enhance consistency of the container; however, as most of these features are meant for scalability, their return on investment is minimal for one-time releases. Thus, these CI/CD tools may be more suited to containers that will be maintained long-term.

It is important to note that we are advocating that researchers consider the cost to benefit ratio of using CI/CD, rather than dissuading researchers from using these tools. Docker Hub and other container archival services can be effectively integrated with GitHub, GitLab, and Bitbucket for automated builds. It should be noted that automated builds can be configured with other types of containers as well. Similar to storage, there are limits on the build or execution time, so containers with numerous dependencies or heavy testing may be unable to use these tools without paying a premium for additional resources (Pricing and Plan Information - CircleCI, 2024; Usage limits, billing, and administration - GitHub Docs, 2024; Pricing | GitLab, 2024b). Additionally, it should be noted that code repository tools with CI/CD capabilities like GitHub and GitLab are not intended for storage, so users should ensure that the final container image is stored on a publicly accessible archival service.

## Supplementary Information

Below is the link to the electronic supplementary material.

Supplementary file1 (DOCX 2392 KB)

## Data Availability

Data used for the supplemental analysis come from the Dallas Lifespan Brain Study, freely available for download from OpenNeuro at https://openneuro.org/datasets/ds004856/versions/1.2.0.
