# Supplementary material for: Enhancing reproducibility in neuroimaging with containerized archival
Source: Brain Imaging Behav. 2026 Aug 24;20(5):123. doi: 10.1007/s11682-026-01191-1 (PMC13503467; doi:10.1007/s11682-026-01191-1)
Supplement: Supplementary file 1 [file 11682_2026_1191_MOESM1_ESM.docx]

Figure S1. Effect size for pairwise differences of FreeSurfer (v7.2.0) hippocampal volume estimates on two different computation environments compared to effect sizes for hippocampal volumes reported in literature^1–3^. For studies where hippocampal volume differences are much larger than processing differences, computation environment differences are not as much of an issue. However, as studies become larger and target smaller effect sizes, accounting for potential computational confounds is essential. Here, we have selected 50 T1-weighted images coming from different participants in the Dallas Lifespan Brain Study (DLBS)^4^. Natively installed FreeSurfer^5^ (v.7.2.0) is run twice on all these images, once on each of two different computers. We compute the pairwise differences in FreeSurfer estimated hippocampal volume between the two different computation environments. Finally, we compute the effect size of the differences and compare to reported effect sizes for hippocampal volume analyses in literature. We note that this analysis is a demonstration of biases that can be introduced when code is not properly containerized. The FreeSurfer pipeline is available to download at: <https://surfer.nmr.mgh.harvard.edu/fswiki/DownloadAndInstall>.

Figure S2. AMD EPYC 9534 CPU - Coefficient of variation (COV) across 50 reruns of the PreQual container on the same input show a low but non-negligible variability (less than 1%) due to the non-determinism of the pipeline. Some voxels, such as those closer to the brainstem or on the edge of the brain, have a much higher variability. The COV was calculated separately for each of the 32 volumes acquired at b1000, and then the COV maps were averaged to obtain the results for the b1000 shell. As only one b0 volume was acquired, no averaging was necessary.

Figure S3. Intel(R) Xeon(R) Gold 6138 CPU - Coefficient of variation (COV) across 50 reruns of the PreQual container on the same input show a low but non-negligible variability (less than 1%) due to the non-determinism of the pipeline. Some voxels, such as those closer to the brainstem or on the edge of the brain, have a much higher variability. The COV was calculated separately for each of the 32 volumes acquired at b1000, and then the COV maps were averaged to obtain the results for the b1000 shell. As only one b0 volume was acquired, no averaging was necessary.

Figure S4. When comparing the distributions of white matter intensity values for each volume averaged across 50 different reruns of the PreQual container, we observe that the two different compute environments have nearly identical distributions for all volumes. This result suggests that variability in the outputs is not coming from the different hardware, but from the inherent nondeterminism of the pipeline.

Figure S5. When researchers are deciding on whether or not to release their method/algorithm as a BIDS-App container, there are several considerations regarding feasibility and usability for the greater neuroimaging community. Even if a container is unsuitable or unable to be released as a BIDS-App, releasing researchers should attempt to provide documentation regarding how end-users can run the container with BIDS-organized data.

Consistency/Verification Guidance

While the specific construction of a neuroimaging container is highly dependent on the underlying algorithms, we recommend the following set of high-level instructions and suggestions for the process of validating and verifying a container.

- **Acquire applicable open-source data for verification**: If researchers have data of the correct input modality available that is openly sharable, they should consider using that as test data for verifying the container. Alternatively, there are many openly available datasets, such as those hosted on OpenNeuro^4^, which can be used as well. Regardless of which data is chosen, releasing researchers of the container should be sure to document and properly cite where the test data originated (e.g. dataset version, specific participant, session, scan, etc.).
- **Validate on test data**: It is important to make sure that the container runs on external data, not only the specific dataset(s) used in the publication or work that the container originated from. Thus, researchers should validate that, provided the properly formatted inputs, the container produces the expected outputs. ***Acceptance criteria***: The pipeline executes from start to finish without errors on the test data.
  - **Assess nondeterminism**: If there are any nondeterministic components of the container, it is important to verify the expected variability due to the nondeterminism. Researchers should at least determine if repeated runs of the container will produce different outputs using the exact same input data, and, if so, should note that in the documentation. For a more in-depth assessment, researchers can rerun the algorithm multiple times and quantify the expected variability. Providing such empirical measurements of variability along with the container release could be beneficial for end users. ***Acceptance criteria***: Level of nondeterminism for outputs is verified – does the container produce different outputs upon a rerun with identical data? For a more in-depth documentation, also provide the expected variability measurements.
- (If needed) **Export the container into acceptable format**: For some archival services, such as Zenodo or OSF, the container must be a single, self-contained file. Other services, such as Dockerhub, require specific container format. Researchers should verify 1.) that the intended archival service supports the container format and 2.) the container is in a format that is supported by the archival service (see Table 2). For the latter, containers such as Docker images that exist in a layered format can be exported to a self-contained file. As an example, if a Docker container is called mycontainer, it can be exported as a single file called container.tar using the command:

docker save -o container.tar mycontainer:latest

Upon downloading the self-contained file, end users can load the Docker for use by running:

docker load -i container.tar

Releasing researchers should make sure that (if applicable) these steps are well-documented for end users. ***Acceptance criteria***: File can be properly uploaded to the selected archival service. Additionally, instructions are provided for loading the container in a usable manner if applicable.

- **Document**: Documentation practices have been extensively covered by the “Documentation” subsection of the Discussion section in the main manuscript. However, below are some useful commands for verification purposes:
  - md5sum and sha256sum: Provide checksums of both containers and test data. It is important to note in the documentation that, for example output data, checksums may not align if containers are nondeterministic when end users are verifying test data.
  - syft: Generates a software bill of materials for a container.

***Acceptance criteria***: Test input and output data for verification purposes, as well as the commands to run the container, should be well documented for end users. Any expected nondeterminism should be noted.

- **Verify the archival**: Following archival of all files (container, test data, etc.) on the intended archival service, redownload the container and test data from the archival service, verifying that any provided tutorial is correct and runs as expected.

[1]	Raz, N., Lindenberger, U., Rodrigue, K. M., Kennedy, K. M., Head, D., Williamson, A., Dahle, C., Gerstorf, D. and Acker, J. D., “Regional Brain Changes in Aging Healthy Adults: General Trends, Individual Differences and Modifiers,” Cerebral Cortex 15(11), 1676–1689 (2005).

[2]	Jäncke, L., Mérillat, S., Liem, F. and Hänggi, J., “Brain size, sex, and the aging brain,” Hum Brain Mapp 36(1), 150–169 (2015).

[3]	Hibar, D. P., Westlye, L. T., Van Erp, T. G. M., Rasmussen, J., Leonardo, C. D., Faskowitz, J., Haukvik, U. K., Hartberg, C. B., Doan, N. T., Agartz, I., Dale, A. M., Gruber, O., Krämer, B., Trost, S., Liberg, B., Abé, C., Ekman, C. J., Ingvar, M., Landén, M., et al., “Subcortical volumetric abnormalities in bipolar disorder,” Mol Psychiatry 21(12), 1710–1716 (2016).

[4]	“The Dallas Lifespan Brain Study - OpenNeuro.”, <https://openneuro.org/datasets/ds004856/versions/1.2.0> (13 January 2025 ).

[5]	Fischl, B., “FreeSurfer,” Neuroimage 62(2), 774–781 (2012).
